# Metabolic Response of *Escherichia coli* upon Treatment with Hypochlorite at Sub-Lethal Concentrations

**DOI:** 10.1371/journal.pone.0125823

**Published:** 2015-05-01

**Authors:** Adrian Drazic, Erika Kutzner, Jeannette Winter, Wolfgang Eisenreich

**Affiliations:** 1 Center for Integrated Protein Science Munich (CiPS^M^), Lehrstuhl für Biotechnologie, Technische Universität München, Lichtenbergstr. 4, Garching, Germany; 2 Lehrstuhl für Biochemie, Technische Universität München, Lichtenbergstr. 4, Garching, Germany; Indian Institute of Science, INDIA

## Abstract

Hypochlorite is a reactive oxygen species that is worldwide as an antibacterial disinfectant. Hypochlorite exposure is known to cause oxidative damage to DNA and proteins. As a response to these effects, the metabolite profiles of organisms treated with sub-lethal doses of hypochlorite are assumed to be severely modified; however, the nature of these changes is hardly understood. Therefore, using nuclear magnetic resonance spectroscopy and gas chromatography-coupled mass spectrometry, we analyzed the time-dependent impact of hypochlorite exposure with a sub-lethal concentration (50 µM) on the metabolite profile of the *Escherichia coli* strain MG1655. Principle component analysis clearly distinguished between the metabolite profiles of bacteria treated for 0, 5,10, 20, 40, or 60 min. Major changes in the relative amounts of fatty acids, acetic acid, and formic acid occurred within the first 5 min. Comparative gas chromatography-coupled mass spectrometry analyses revealed that the amounts of free methionine and alanine were significantly decreased in the treated cells, demonstrating their susceptibility to hypochlorite exposure. The concentrations of succinate, urea, orotic acid, 2-aminobutyric acid, and 2-hydroxybutyric acid were also severely affected, indicating general changes in the metabolic network by hypochlorite. However, most metabolite levels relaxed to the reference values of untreated cells after 40–60 min, reflecting the capability of *E*. *coli* to rapidly adapt to environmental stress factors such as the presence of sub-lethal oxidant levels.

## Introduction

Reactive oxygen species (ROS) are formed as by-products of aerobic respiration. In addition, they are generated by the innate immune system and mucosal barrier epithelia to kill invading bacteria [1, 2]. Uncontrolled myeloperoxidase-driven overproduction of ROS in activated neutrophils and macrophages is associated with a series of diseases such as cancer, chronic inflammation, pulmonary, and neurodegenerative diseases [2, 3]. Furthermore, ROS accumulate during aging and cause age-related oxidative stress symptoms [4, 5]. ROS differ in their reactivity with macromolecules and thus in their antimicrobial activity. Most ROS such as the superoxide anion and hydrogen peroxide (H_2_O_2_) react rather selectively with macromolecules [6]. Hypochlorite (HOCl) is even more reactive and bactericidal and is thus used widely as a disinfectant in hospitals and households (commonly known as “bleach”) [7]. HOCl reacts with iron ions via the Fenton reaction, forming cytotoxic hydroxyl radicals [8, 9]. Furthermore, HOCl generates toxic compounds such as methylglyoxal and reactive electrophiles [10]. HOCl targets and damages various macromolecules, including DNA, lipids, and proteins. This eventually leads to genome-wide mutations, protein inactivation, and perturbations in membrane function. In contrast to H_2_O_2_, which specifically leads to cysteine oxidation, HOCl treatment causes oxidative unfolding of proteins [11, 12]. Besides, it is reactive toward the sulfur-containing side chains of the amino acids methionine and cysteine, causing oxidation to methionine sulfoxide (Met-SO) or even methionine sulfone as well as the formation of disulfide bonds and oxidation to keto acids (e.g., sulfenic, sulfinic, and sulfonic acid), respectively [11]. Notably, only the mono-oxidized modifications such as methionine sulfoxide and sulfenic acid can be reduced back to their prior state by the action of specific reductases such as methionine sulfoxide reductases and thioredoxins, respectively [13].

Several studies have shown that HOCl causes the induction of heat shock and SoxR (transcription factor for superoxide detection) responses [14]. In addition, it has been shown that HOCl stress causes global thiol oxidation [15] as well as proteome-wide unfolding and aggregation [16]. Proteome-wide analysis of methionine modifications under HOCl exposure showed a massive increase in methionine oxidation to methionine sulfoxide and sulfone [13, 17]. In contrast, recent studies have revealed that several proteins such as the transcription factors HypT, NemR, and RclR or the chaperone Hsp33 become activated by HOCl apart from the damage caused to proteins [10, 16, 18–21]. Importantly, all these proteins use the normally destructive post-translational modifications such as oxidative unfolding and, cysteine and methionine oxidation to initiate their activation [13, 18, 22].

To further understand the mechanisms underlying ROS action and the resulting bacterial responses, investigations on the transcriptional and proteomic levels are not sufficient, and these have to be accompanied by analysis of the metabolite profiles. Metabolite profiles allow conclusions to be drawn on precise cellular responses resulting in HOCl-induced metabolic alterations. Focusing on the metabolic effects upon exogenous stress factors, the changes in the metabolite profiles of *E*. *coli* due to heat-, cold-, and H_2_O_2_-induced oxidative stress were compared [23]. Several metabolites were identified as general “stress responders,” independent of the type of stress they were exposed to.

In the present study, we aimed to analyze the time-dependent metabolic responses of *E*. *coli* by nuclear magnetic resonance spectroscopy (^1^H-NMR) and gas chromatography-coupled mass spectrometry (GC/MS) upon 5–60 min treatments with sub-lethal HOCl doses. ^1^H-NMR spectra of cell extracts were analyzed by principal component analysis (PCA) in addition to specific metabolite identification by GC/MS in an attempt to describe time-dependent global and specific HOCl-induced effects on the metabolite composition of *E*. *coli*. Our study revealed that major changes in the metabolite profile occur as early as 5 min of HOCl-induced stress. These included major shifts in the relative concentrations of fatty acids, amino acids, and other organic acids such as acetic acid and formic acid. In addition, after 40–60 min, we observed the regeneration of several metabolites to levels comparable to those observed before HOCl exposure. This quick adaptation of *E*. *coli* to exogenous stress factors demonstrates its evolutionary ability to survive under harsh environmental conditions and habitats.

## Materials and Methods

### Strains, media, and growth conditions

*E*. *coli* MG1655 was aerobically grown at 37°C in M9 minimal medium (Na_2_HPO_4_·2 H_2_O, 7.1 g L^−1^; KH_2_PO_4_, 3 g L^−1^; NaCl, 0.5 g L^−1^; NH_4_Cl, 1 g L^−1^), supplemented with 2 mL 1 M MgSO_4_, 20 mL 20% (w/v) D-glucose, and 1 mL 0.1 M CaCl_2_ per liter.

### Analysis of bacterial viability

For bacterial viability experiments, *E*. *coli* MG1655 cells were cultivated in M9 minimal media until an A_600_ of 0.4–0.5 was reached. Following this, the cells were washed twice with 0.9% (w/v) sodium chloride buffer and re-suspended in fivefold of the original volume of M9 minimal medium. The washed cells were distributed into 15-mL tubes (1 mL each), following which HOCl (Sigma-Aldrich; final concentration 0–75 μM in 25 μM steps) was added. In total, 1 mL of fivefold-concentrated Luria–Bertani (LB) medium was added at the different time points to quench the HOCl stress [16]; cells were serially diluted in LB medium (1:30 dilutions) and spotted onto LB agar plates.

### Metabolite extraction

Cells were inoculated from a glycerol stock and cultivated overnight at 37°C (shaking at 160 rpm) in 150 mL M9 minimal medium. After 16 h, cells were diluted to 1:20 and cultivated at 37°C (shaking at 160 rpm) in freshly prepared M9 minimal medium until an A_600_ of 0.4–0.5 was reached. Following this, cells remained untreated (control) or were stressed by adding 50 μM HOCl under short vortexing. At intervals (5, 10, 20, 40, or 60 min), an equivalent of the suspension was removed and immediately centrifuged (5000 rpm, 5 min, 4°C) to obtain the required amount of cell material for the NMR experiments. Cell, pellets were immediately frozen in liquid nitrogen to stop metabolic reactions. For these experiments, no LB medium was used to stop HOCl stress for ensuring that all detected metabolites were derived from the *E*. *coli* cells and not from the media. The frozen cell pellets were dried by lyophilization. In total, 45–55 mg of dried cell material was re-suspended in 1 mL of D_4_-methanol, and an approximate volume of 500 μL of glass beads (0.25–0.5 mm) was added. Cells were disrupted by three 20-s steps (6.5 m/s) in a FastPrep FP 120 Cell Disrupter (Qbiogene, Inc.). Cell debris and glass beads were removed by centrifugation [7000 rpm, 7 min, room temperature (RT)], and the supernatant was used for NMR and GC/MS analysis (S1 Table).

### NMR acquisition

For NMR analysis, 600 μL of the methanol extract (D_4_-methanol) was transferred into a 5-mm NMR tube. All experiments were performed at 300 K using the Avance I 500 System (Bruker Biospin, Rheinstetten, Germany) with an UltraShield 500 MHz magnet and a SEI 500 S2 probe head (5 mm, inverse ^1^H/^13^C with Z-gradient). The measurements were conducted at a magnetic field of 11.75 T. The resonance frequency of ^1^H was 500.13 MHz. For all samples, the ^1^H-NMR spectra were acquired using the one-dimensional NOESY sequence “noesygppr1d.comp” with presaturation of the residual water signal during the relaxation delay and the mixing time using spoil gradients. The relaxation delay was 4.0 s, and the acquisition time was 3.3 s. Spectra were the result of 128 scans, with data collected into 64 k data points. Each FID was zero-filled to 128 k data points. Prior to Fourier transformation, an exponential window function with a line broadening factor of 0.2 Hz was applied. The resulting spectra were manually phased and baseline corrected using TopSpin 3.1 (Bruker Biospin, Rheinstetten, Germany) and referenced to 0.1 μM internal trimethylsilyl-2,2,3,3-tetradeuteropropionic acid sodium salt (TSP) at 0.0 ppm.

### GC/MS sample preparation and data analysis

In total, 5 μL of 5 mM D-norvaline (internal reference) was added to 600 μL of the methanolic extract. The mixture was dried under N_2_ flow at RT. The residue was dissolved in 200 μL of pyridine containing 20 mg mL^−1^ methoxyamine hydrochloride and incubated at 40°C for 1.5 h (shaking at 200 rpm). The reaction mixtures were again dried under N_2_ flow and then treated with 100 μL N-methyl-N-(trimethylsilyl)-trifluoroacetamide (with 1% trimethylchlorosilane; MSTFA) at 50°C for 30 min(shaking at 200 rpm). These samples were directly subjected to GC/MS analysis. The GC/MS measurements were performed on a GC/MS-QP 2010 Plus mass spectrometer coupled to a QP-5000 mass selective detector (Shimadzu, Duisburg, Germany) working with electron impact (EI) ionization at 70 eV and scanning from *m/z* 50 to *m/z* 800. A Silica capillary column Equity TM-5 (30 m × 0.25 mm × 0.25 μm film thickness) from Supelco Inc. (Bellefonte, PA, USA) was used. An aliquot of derivatized metabolites was injected in a 1:10 split mode at a temperature of 260°C and a helium inlet pressure of 76.1 kPa. The interface temperature was 260°C, and the helium column flow was 1.19 mL/min. The column was developed at 70°C for 5 min and then with a temperature gradient of 5°C/min to a final temperature of 300°C, holding for 2 min. The system was provided with GC/MS solution Ver. 2 software, and identification of the metabolites was performed using the NIST05 and NIST05s mass spectral reference library [24].

### Data analysis

For data analysis, 98 ^1^H-NMR spectra (S1 Table) were imported into AMIX (Analysis of Mixtures software package, version 3.9.13, Bruker Biospin, Rheinstetten, Germany) and subjected to PCA. The 98 spectra included measurements of methanolic extracts of *E*. *coli* cells subjected to six different treatments, namely no HOCl stress (control group) and HOCl stress for 5, 10, 20, 40, and 60 min. For detailed sample listing, see Supporting Information S1 Table. Spectra were divided into equally sized bins with a width of 0.05 ppm within the region of 10.0 ppm to 0.2 ppm using the advanced bucketing command in AMIX software. Each bin was then integrated, and a data matrix was constructed with each row representing a spectrum and each column representing a variable (i.e., bin). Rows were scaled to the total intensity of the spectrum, and the resulting values were used for PCA. The spectral regions between 4.9–4.8 ppm, 3.40–3.25 ppm, and 1.21–1.14 ppm were excluded in PCA analysis. PCA was performed with no scaling of columns using AMIX software.

## Results

### Determination of sub-lethal HOCl concentrations for *E*. *coli*

**Fig 1:**
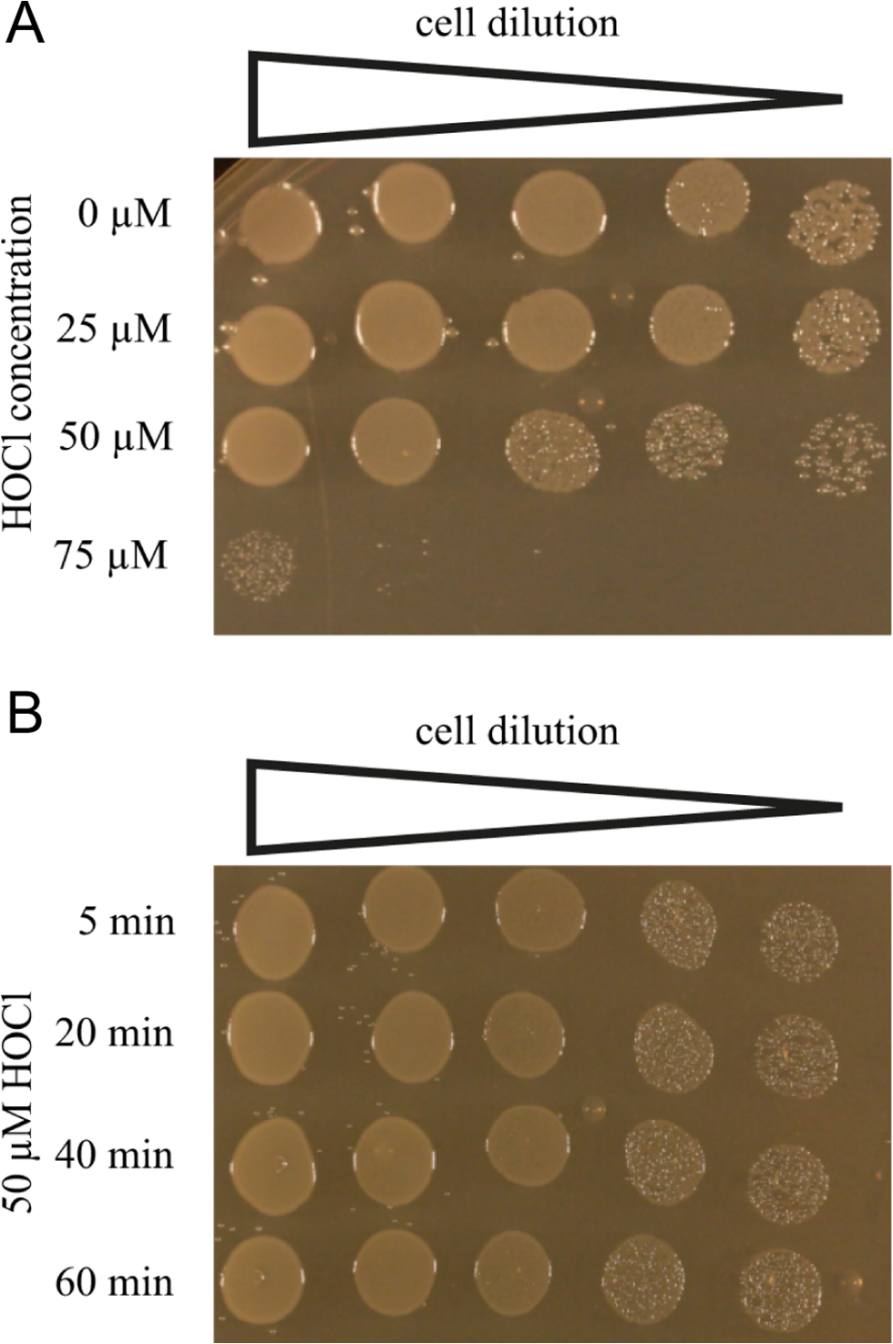
Identification of a sub-lethal HOCl concentration. Viability of *E*. *coli* MG1655 cells was analyzed in M9 minimal medium in the presence of the indicated concentrations of HOCl. Samples were removed after 10 min (A) or the indicated time points (B), serially diluted to 1:30, and spotted onto LB agar plates (1st to 5th dilution). (A) The viability was analyzed in 25 μM HOCl concentration steps. Cells were 100% viable at 0 and 25 μM HOCl. At 50 μM HOCl, they showed first indications of cell stress. Nevertheless, the cell viability remained at a high level. At 75 μM HOCl, the cells are harshly stressed and show a very low viability. (B) Cells were treated with 50 μM HOCl for various time points. The survival assay indicates cell stress; however, the viability remains high even after 60 min of HOCl treatment. The figure shown is the result of one representative experiment.

To detect alterations in metabolite levels and determine the response of *E*. *coli* encountering HOCl stress, we tested various HOCl concentrations and analyzed the viability of cells grown in M9 minimal medium. The objective was to identify a sub-lethal HOCl concentration that enables *E*. *coli* cells to actively respond to this stress factor and to potentially compensate the bactericidal effects of HOCl exposure by metabolic adaptations. We cultivated *E*. *coli* MG1655 cells in M9 minimal medium to an A_600_ of 0.5 and treated the population with HOCl in 25 μM increments (25–75 μM) for 5–60 min. HOCl stress was quenched by the addition fivefold-concentrated LB medium [16, 25]. Following this, the cells were serially diluted (1:30) in LB medium and spotted onto LB agar plates (Fig 1). Cells treated with HOCl concentrations below 50 μM HOCl showed a viability of 100% (Fig 1A). First indications for stressed cells occurred at 50 μM HOCl. Nevertheless, the cells still showed a high viability. At higher HOCl concentrations (75 μM), the viability of the cells significantly decreased. Following this, we analyzed the longevity of cells treated with 50 μM HOCl. We stopped the HOCl reaction after 5, 20, 40, and 60 min, showing that cells remained viable and were not killed by 50 μM HOCl exposure even after 60 min (Fig 1B).

### Determination of global alterations in metabolite profiles by NMR and PCA

**Fig 2:**
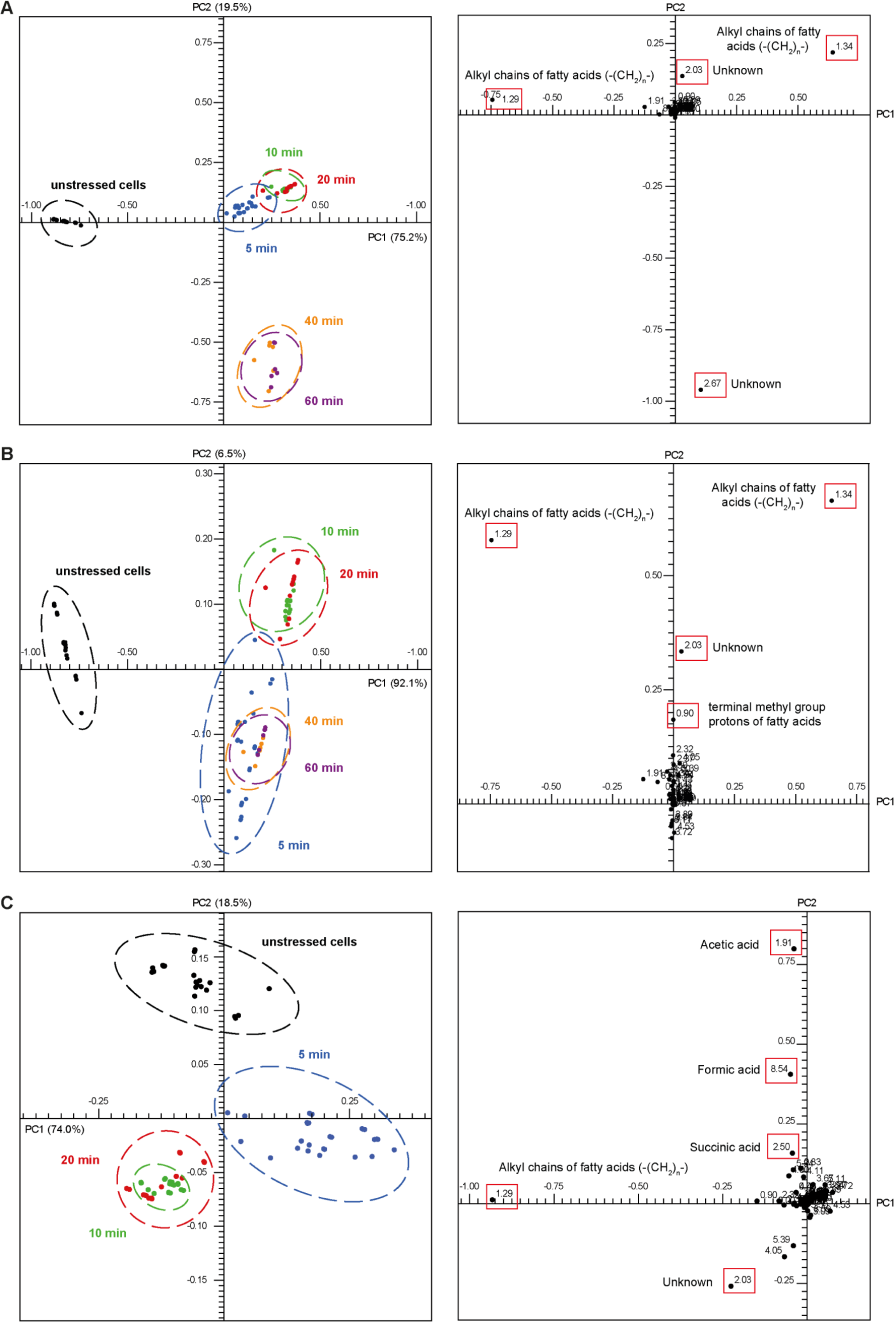
Principle component analysis of ^1^H-NMR spectra of unstressed and HOCl-stressed *E*. *coli* cell extracts. (A) PCA scores plot (on the left side) and loadings plot (on the right side) derived from ^**1**^H-NMR data of methanol extracts of unstressed *E*. *coli* MG1655 cells (black dots) and cells stressed for 5 min (blue dots), 10 min (green dots), 20 min (red dots), 40 min (orange dots),) and 60 min (purple dots). Each dot in the scores plot represents a certain ^**1**^H-NMR spectrum (S1 Table). Each point in the loadings plot represents a spectral region (bin) on the scale of 0.05 ppm. Loadings highlighted in red boxes mark the ^**1**^H-NMR peaks that were responsible for the greatest variance in the data and therefore for the group building in the scores plot. Scores and loadings for PC1 against PC2 are shown. Percentages in brackets specify the variance within all samples explained by a PC. (B) Recalculation of the PCA presented in Fig 2A by applying the same parameters and spectra; the bin at 2.67 ppm was not considered (see text for more details). (C) PCA scores plot (on the left side) and loadings plot (on the right side) derived from ^**1**^H-NMR data of methanol extracts of unstressed *E*. *coli* MG1655 cells (black dots) and cells stressed for 5 min (blue dots), 10 min (green dots),) and 20 min (red dots). By focusing on the early stress response of *E*. *coli*, the loadings plot revealed additional outliers that contributed to the group formation in the scores plot.

To investigate global alterations in metabolite levels after HOCl stress, *E*. *coli* cells were exposed to 50 μM HOCl for 5, 10, 20, 40, and 60 min, and harvested by rapid centrifugation. The pellets were treated with liquid nitrogen to quench metabolic reactions. The frozen pellets were dried by lyophilization and subsequently disrupted and extracted with D_4_-methanol in a ribolyser. After centrifugation, the methanolic supernatants were immediately subjected to ^1^H-NMR analysis. Typical ^1^H-NMR spectra of extracts from stressed and unstressed cells are shown in S1 Fig. Visual inspection revealed differences between these spectra. For example, extracts from cells stressed for 5, 10, 20, 40, and 60 min did not display peaks at 8.5 ppm, in contrast to the reference spectrum. Differences in peak intensities could also be observed around 1.9 ppm. Because the visual inspection of 98 ^1^H-NMR spectra (see S1 Table and Materials and Methods section) measured within the framework of these experiments is not useful, all spectra were digitized and statistically analyzed by PCA (see Materials and Methods). The global changes in the metabolic profiles were visualized as a two-dimensional PCA scores plot (Fig 2A on the left side). The plot is composed of PC1 (75.2%, x-axis) and PC2 (19.5%, y-axis), explaining 94.7% of the total variance within all samples. Black, blue, green, red, orange, and purple circles indicate spectra derived from unstressed cells and cells stressed for 5, 10, 20, 40, and 60 min, respectively. It must be emphasized that some individual bacterial samples (i.e., biological replicates) in the scores plot were represented by three circles (i.e., three spectra) as they were measured three times (S1 Table). Measurements of the same sample clustered very closely in the scores plot, indicating the high stability of the ^1^H-NMR measurements. Spectra derived from biological replicates showed greater grouping variance. Nevertheless, defined clusters could be observed for each experimental condition (i.e., unstressed; stressed for 5, 10, and 20 min; and stressed for 40 and 60 min).

In PC1 direction, a clear separation between unstressed cells (black circles) and stressed cells could be observed. Data from unstressed cells were identified as a separate group along the negative axis of PC1, whereas data from the stressed cells were located in the positive area of PC1. Furthermore, clustering within the different periods of HOCl stress was evident on the basis of PC2. Spectra derived from cells stressed for 5, 10, and 20 min with HOCl (blue, green, and red circles, respectively) grouped together in the positive side of the first Cartesian quadrant. Within this group, spectra recorded after 5 min of stress (blue circles) were slightly separated from those recorded after 10 and 20 min of stress (green and red circles, respectively). Cells stressed for 40 min (orange circles) and 60 min (purple circles) were located at the negative side of the PC2 axis and could not be distinguished from each other. *E*. *coli* cells stressed for 10 and 20 min clustered as well and gave rise to a very tight group, which implies very similar NMR spectra and a similar metabolite composition of those samples. Therefore, every group (i.e., every stress condition) led to a specific metabolic composition, which is different from the other stress conditions. The grouping behavior points to a very quick response of the cells to HOCl stress. Within the first 5 min of HOCl stress, the metabolite profile was subject to significant changes in comparison with the reference spectra. Within the next 15 min, when comparing the 5 min and the 20 min samples, these alterations decreased to a minimum (e.g., only small changes according to PCA). The group reflecting cells stressed for 40 and 60 min was separated from the control group as well as from the other stress conditions, indicating significant metabolic changes. However, the grouping of cells stressed for 40 and 60 min, which were far apart from the other groups, arose only from one signal, which is located at 2.67 ppm because of the loadings plot (Fig 2A on the right side). This singlet could not be assigned to a specific metabolite and it is absent in all spectra derived from the other stress conditions. In addition, its intensity is 10-fold higher than the intensity of all other signals. Such a big difference in intensities could lead to misinterpretations in the PCA because alterations occurring at signals with a low intensity would be underestimated. Consequently, we performed the PCA again by excluding this signal (Fig 2B). Fig 2B (on the left side) displays the recalculated scores for all stress conditions and reveals a more conclusive grouping behavior. The unstressed cells were still located along the negative axis of PC1, and the stressed cells were located on the positive side of the same axis. Cells stressed for 10 and 20 min remained on the positive side of PC2; however, cells stressed for 5 min could be found in the negative area of PC2 now, grouping together with cells stressed for 40 and 60 min. On the one hand, an unambiguous separation of cells stressed for 5 min and for 10 and 20 min is evident, which implies metabolic changes within this time. On the other hand, cells stressed for 5, 40, and 60 min built up a separate group, which implies similar spectral profiles and thus metabolic alterations between 10/20 min and 40/60 min. The similar metabolic profiles of cells stressed for 5 min and cells stressed for 40 and 60 min indicate a regeneration of the stressed *E*. *coli* cells toward the metabolic state of unstressed cells (i.e., position of black circles in the scores plot).

To shed light on the early stress response of *E*. *coli*, PCA was calculated by omitting spectra measured after 40 and 60 min of HOCl stress. Fig 2C shows the scores plot for unstressed cells and cells stressed with HOCl for 5, 10, and 20 min on the left side. This analysis confirmed the grouping behavior determined in the previous PCA (Fig 2B), which exhibited a clear separation of unstressed cells from stressed cells as well as of cells stressed for 5 min from cells stressed for 10 and 20 min. In addition, this recalculation revealed additional bins responsible for the group formation according to the loadings plot (Fig 2C on the right side).

In conclusion, the global metabolic profile was highly dynamic during 60 min HOCl exposure, and the stressed cells were not able to entirely reconstruct the metabolic state of unstressed cells within 60 min. However, an approximation from stressed cells to the metabolic state of unstressed cells could be observed, as indicated by the location of spectra recorded after stress application of 5 min and spectra derived after 40 and 60 min of stress at the same area in the scores plot (Fig 2B). This is in agreement with the GC/MS findings; most metabolite levels were altered after 20 min of HOCl stress in comparison with the levels detected in unstressed cells but converged in most cases toward the unstressed state after 60 min (as mentioned below).

### 1H-NMR peak assignments

To identify the signals responsible for the clustering in the scores plot, the corresponding loadings were analyzed. In Fig 2 on the right side, the two-dimensional loadings plots for PC1 against PC2 are shown. Each point in the loadings plot represents a spectral section (i.e., bin) on the scale of 0.05 ppm. The position of a certain bin in the loadings plot corresponds to the group of samples located at the same position in the scores plot. The value of a loading represents the importance of the corresponding spectral region for the clustering in the scores plot. For instance, the influence of the bin at 1.91 ppm (Fig 2C) on the grouping in the scores plot is greater than that of the bin at 8.54 ppm as it possesses a higher PC2 value in the loadings plot (0.8 and 0.4 on the y-axis, respectively).

By examining the most influential bins, spectral regions became apparent, which caused the separation in the scores plot between the unstressed samples of *E*. *coli* and the samples exposed to HOCl. The most prominent outlier was the bin at 2.67 ppm located at the negative end of the PC2 axis (Fig 2A), which was excluded from further analyses, as explained in the previous section. The remaining bins of high value, shown in Fig 2B on the right side, were bins at 1.29 ppm and at 1.34 ppm. The bin at 1.29 ppm was a very strong outlier at the negative side of PC1, whereas the bin at 1.34 ppm could be found at the positive side of the same axis. These spectral regions were responsible for the separation of the unstressed cells (black circles positioned at the negative side of PC1) from the stressed cells (located at the positive side of PC1). Both adjacent bins mainly contained a broad signal originating from long-chain fatty acids (Table 1). On the positive side of the PC2 axis (Fig 2B), the bin at 2.03 ppm was detectable as an outlier. Compared with the samples stressed for 5, 40, and 60 min or unstressed samples, the signals in this bucket were of a greater intensity in spectra derived from samples stressed for 10 and 20 min. However, because of potential signal overlapping, this bin could not be assigned unequivocally to a specific metabolite. An additional prominent bin along the positive PC2 axis was the signal region at 0.90 ppm whose signals could be caused by the terminal methyl protons of fatty acids (Table 1).

**Table 1:** Reference metabolites measured by ^1^H-NMR spectroscopy using MeOD as solvent.

Metabolite	Chemical shifts [ppm] and multiplicity
Alanine	1.46 (d), 3.58 (q)
D-Glucose	3.13 (dd), 3.31 (m), 3.36 (dd), 3.68 (m), 3.78 (m), 3.85 (dd), 4.48 (d), 5.11 (d)
Glutamine	2.09 (m), 2.46 (m), 3.58 (t)
Lactic acid	1.32 (d), 3.98 (q)
Methionine	2.06 (m), 2.11 (s), 2.63 (m), 3.66 (dd)
Methionine sulfoxide	2.27 (m), 2.67 (s), 2.99 (m), 3.64 (m)
Palmitic acid	0.90 (t), 1.29 (m), 1.60 (m), 2.27 (t)
Succinic acid	2.49 (s)

s—singlet, d—doublet, dd—doublet of doublets, t—triplet, q—quartet, m—multiplet

Metabolites were measured under the same conditions as bacterial samples and referenced to TMS.

To provide more information on the early stress response of *E*. *coli*, PCA was recalculated by excluding spectra recorded after 40 and 60 min of HOCl stress (Fig 2C). The resulting loadings plot (Fig 2C on the right side) revealed further outliers, which contributed to the group formation in the scores plot after short periods of HOCl stress. On the positive side of the PC2 axis, the bin at 1.91 ppm acted now as a strong outlier (Fig 2C on the right side). This spectral region apparently refers to the ^1^H-NMR methyl signal of acetate [26], and indeed, this signal showed a higher intensity in the control samples than in samples from HOCl-stressed cells. The same was true for the spectral region at 8.55 ppm, referring to formic acid [26], which could be found as an outlier on the positive side of the PC2 axis as well. A less prominent outlier along the PC2 axis was the bin at 2.50 ppm. This signal was tentatively assigned as succinate (Table 1). Its peak intensity increased to a maximum after 20 min of HOCl stress and decreased immediately thereafter. The signal intensity of succinate diminished to its initial level after 40 min of stress application. These findings were verified by GC/MS analysis (see subsequent section). The bin at 1.29 ppm, containing fatty acid signals, was again a very strong outlier at the negative side of PC1. Compared with all other samples in this bin, samples from 5 min HOCl exposure displayed a decreased signal in the NMR spectra. From these findings, it can be inferred that the content of free fatty acids was reduced in the cells shortly after HOCl exposure. However, the 1.29 ppm bin recovered very quickly. After 10 min, its intensity was already as high as that in the control samples. The intensity remained at the control level after 20 min of HOCl stress. This was in good agreement with the GC/MS data for fatty acids (as mentioned below).

In summary, PCA revealed that unstressed cells mainly differ from 5 min HOCl-stressed cells in their acetate and formic acid contents, which are significantly higher without stress. In addition, a significant alteration in fatty acid content was observable. NMR-based PCA led to a clear distinction between the unstressed cells and cells stressed with HOCl for different periods of time.

### GC/MS analyses of HOCl-stressed cells

**Fig 3:**
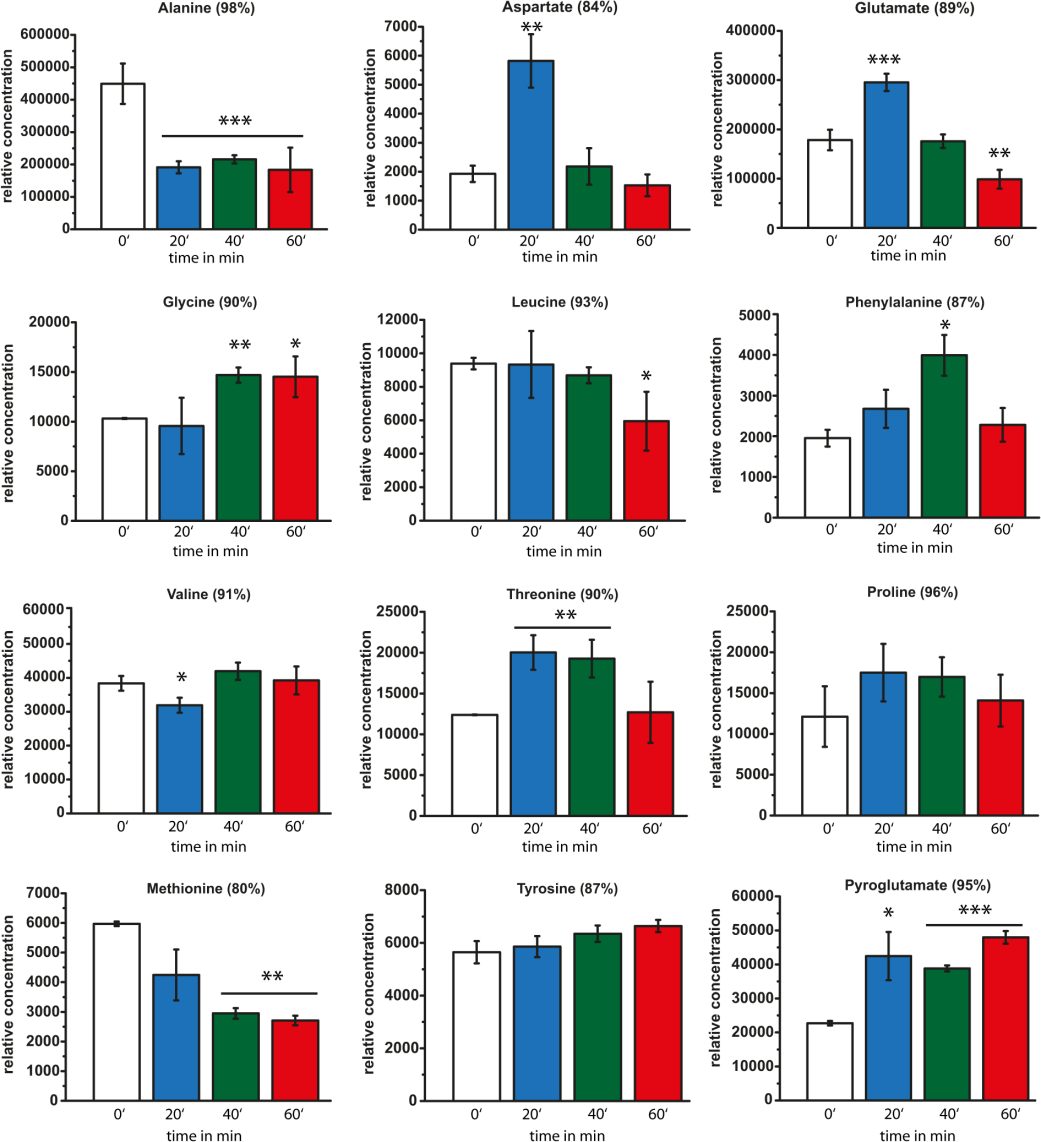
Time-dependent GC/MS analysis of amino acid levels of unstressed and HOCl stressed cells. Relative concentrations of amino acids identified by GC/MS analysis prior to HOCl stress (white bars), and cells treated with 50 μM HOCl for 20 min (blue bars), 40 min (green bars), and 60 min (red bars). Concentrations are normalized to dry cell mass. Hit percentage of detected compounds compared with the NIST05 and NIST05s library is indicated in brackets after the compound name. Statistical significances are indicated as asterisks determined by Student’s t-test: *p < P<0.05; **p < P<0.01; and ***p < P<0.001. Shown is the mean ± standard deviation (n ≥ 3).

**Fig 4:**
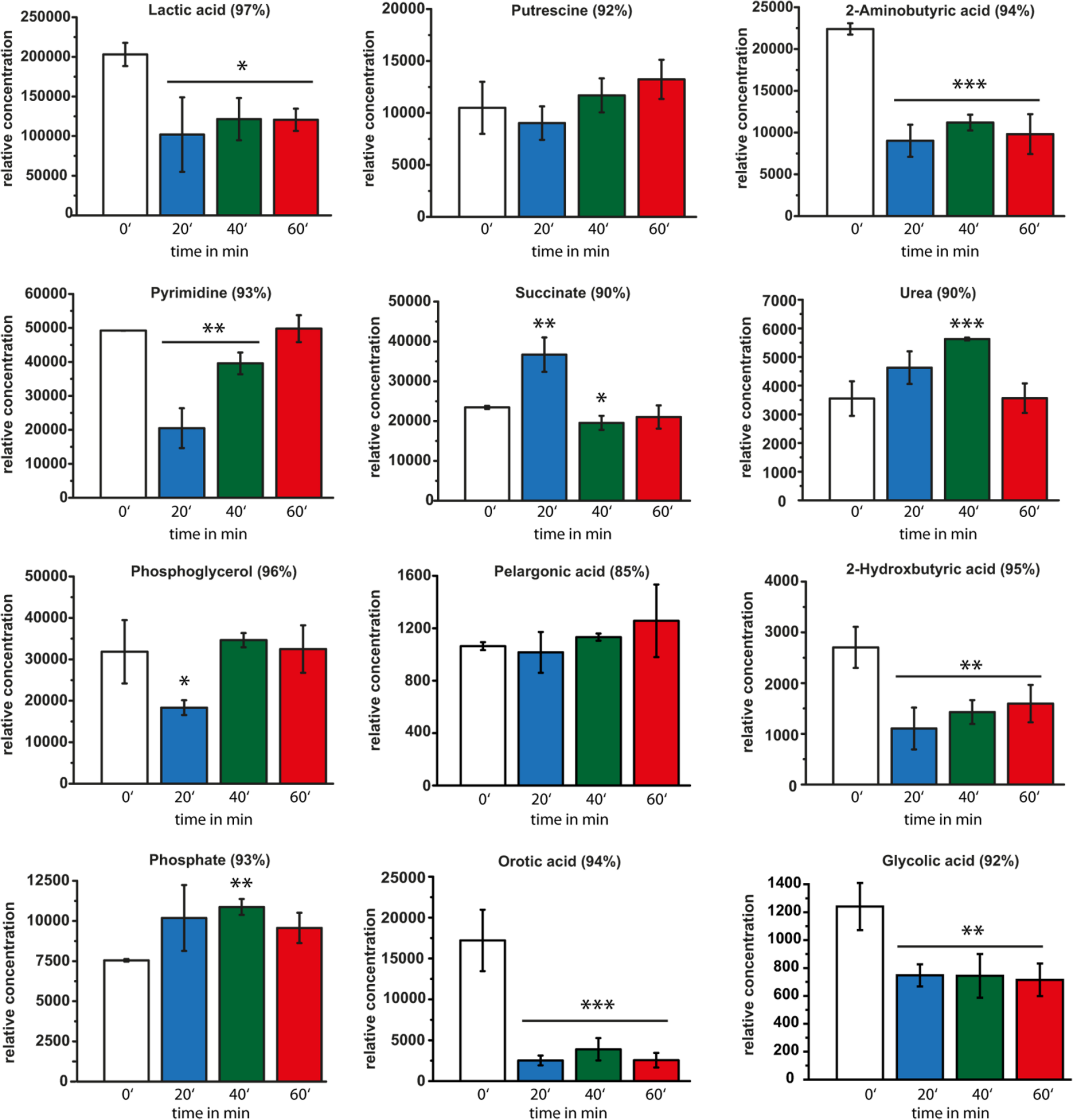
Time-dependent GC/MS analysis of metabolites involved in various metabolic pathways. Relative concentrations of various metabolites involved in different metabolic pathways such as phosphate metabolism, organic acid metabolism and amine metabolism, identified by GC/MS analysis prior to HOCl stress (white bars) and in cells treated with 50 μM HOCl for 20 min (blue bars), 40 min (green bars), and 60 min (red bars). Concentrations are normalized to dry cell mass. Hit percentage of detected compounds compared with the NIST05 and NIST05s library is indicated in brackets after the compound name. Statistical significances are indicated as asterisks determined by Student’s t-test: *p < P<0.05; **p < P<0.01; and ***p < P<0.001. Shown is the mean ± standard deviation (n ≥ 3).

**Fig 5:**
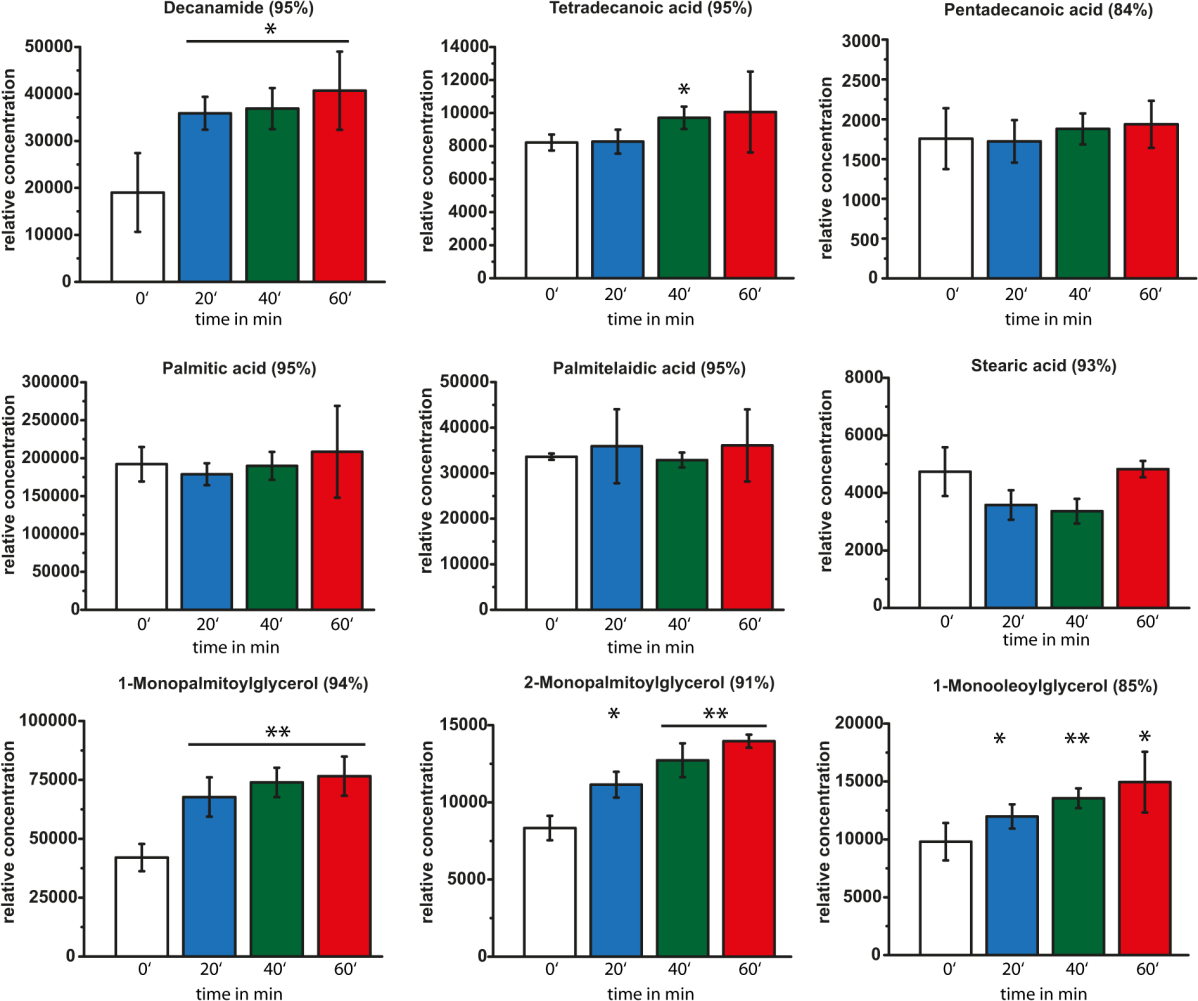
Time-dependent GC/MS analysis of metabolites involved in fatty acid metabolism and membrane integrity. Relative concentrations of metabolites involved in fatty acid metabolism and membrane integrity identified by GC/MS analysis prior to HOCl stress (white bars) and in cells treated with 50 μM HOCl for 20 min (blue bars), 40 min (green bars), and 60 min (red bars). Concentrations are normalized to dry cell mass. Hit percentage of detected compounds compared with the NIST05 and NIST05s library is indicated in brackets after the compound name. Statistical significances are indicated as asterisks determined by Student’s t-test: *p < P<0.05; **p < P<0.01; and ***p < P<0.001. Shown is the mean ± standard deviation (n ≥ 3).

In addition to analyzing the alterations in metabolite levels by ^1^H-NMR fingerprint analysis, we examined metabolite profiles by GC/MS. We arranged the identified metabolites in three different groups belonging to the families of amino acid metabolism (Fig 3), organic acids (Fig 4), and metabolites involved in fatty acid/lipid metabolism and membrane integrity/structure (Fig 5). An example of a typical GC run is shown in S2 Fig The retention times and typical m/z peaks of detected compounds, which could be assigned to specific metabolites via the NIST05 and NIST05s compound library, are summarized in S2 Table. S2 Table also indicates the hit percentage and therefore the reliability of the m/z data related to the NIST05 and NIST05s library. In addition, S3 Table shows all identified peaks and relative concentrations at the different time points of HOCl exposure. However, many of these peaks could be assigned on the basis of reference spectra in the used spectral libraries herein (NIST05 and NIST05s). The following discussion is focused on those peaks that displayed different concentrations of the corresponding metabolites because of HOCl treatment and peaks that could be assigned with a hit percentage of at least 80% by comparison with reference spectra.

### Amino acid metabolism is significantly altered by HOCl stress

By analyzing free amino acids, we observed a decrease in alanine upon HOCl exposure (Fig 3). The alanine level decreased after 20 min to 42% of the original level prior to HOCl stress. These levels remained low after longer time periods of HOCl treatment (48% after 40 min; 40% after 60 min). We observed a similar behavior for methionine, whose levels constantly decreased over time to 71% (20 min), 49% (40 min), and 45% (60 min) (Fig 3). For both amino acids, an antioxidative effect was shown upon H_2_O_2_ and HOCl stress [27, 28]. Methionine seems to react, particularly with various ROS, thus becoming oxidized. This directly results in a quenching of stress and cell damage [13, 28]. Our observed HOCl stress-related decrease in alanine and methionine was in sharp contrast to the dynamic behavior of other detected amino acids. More specifically, most other amino acids showed increased levels after stress exposure in line with previous studies, in which cells were treated with H_2_O_2_ or kept in a prolonged nongrowing phase [23, 29]. For example, aspartate and glutamate showed a threefold and almost twofold increase (302% for aspartate; 165% for glutamate), respectively, after 20 min of HOCl treatment. Notably, both amino acids attained their original levels prior to HOCl exposure as early as 40 min after and did not change these levels after longer HOCl treatments. This is similar to the determined levels of phenylalanine, valine, threonine, and proline (Fig 3). The levels of threonine and proline also increased after HOCl stress (161% after 20 min for threonine; 144% after 20 min for proline) and were maintained after 40 min, until they reached concentrations comparable with those in the unstressed cells. The phenylalanine levels increased at a constant rate until 40 min (136% after 20 min; 204% after 40 min). Further exposure (60 min) resulted in normalization to reference levels (Fig 3). Other amino acids, such as leucine and tyrosine, did not significantly alter their concentrations upon HOCl stress. Glycine did not show altered levels until 40 min of HOCl stress, which resulted in a 142% increase. The level of the amino acid derivative pyroglutamic acid increased twofold after 20 min of HOCl exposure and remained constant at this high level. Pyroglutamic acid is a breakdown product of the glutathione cycle, which could play an important role in the defense of cells against oxidative stress [30]. It has been shown that four HOCl molecules can be sequestered by one molecule of glutathione [31]. Consequently, high pyroglutamic acid levels could result from this sequestration and degradation of glutathione. Otherwise, it is also conceivable that upon the harsh stress conditions, spontaneous cyclization of free glutamate and glutamine occurs, resulting in the increased pyroglutamate levels [32]. However, the second hypothesis appears less probable because glutamate levels also increased after 20 min of HOCl exposure.

### Alterations in metabolites belonging to the families of organic acids, phosphates, and amines

We observed a decrease in lactic acid (50% after 20–60 min) (Fig 4). The same results were obtained when determining the concentrations of 2-aminobutyric acid (40% after 20 min), 2-hydroxybutyric acid (40% after 20 min), and glycolic acid (58% after 20 min), respectively. An almost fourfold decrease was detected for orotic acid. Other metabolites such as phosphoglycerol and succinate rapidly changed their levels after HOCl exposure (58% for phosphoglycerol; 156% for succinate after 20 min); however, both relaxed after 40 min to the levels observed in control cells. The levels of urea increased after HOCl stress (130% after 20 min; 158% after 40 min), reaching the maximum after 40 min (Fig 4). After 60 min of HOCl treatment, it relaxed to the concentration of urea in unstressed control cells. Free phosphate seemed to slightly increase upon HOCl stress (134% after 20 min; 144% after 40 min) but slowly decreased again after 60 min (126%). The regulation of the free phosphate levels could be directly or indirectly involved in a manner described for the function of pyrophosphate as a chaperone and its regulation involved in stress [33]. In contrast, pelargonic acid (nonanoic acid) seemed to remain at a constant level (Fig 4). Another interesting behavior involved pyrimidine. We observed a 59% decrease in pyrimidine levels after 20 min of HOCl stress, which slowly but constantly reached the reference levels of untreated cells (80% after 40 min; 101% after 60 min). Furthermore, we observed that putrescine (1,4-diaminobutane) was not significantly altered during HOCl stress (Fig 4). On this basis, the *E*. *coli* response to HOCl seems to differ from that toward H_2_O_2_ because putrescine was shown to play a major role in the regulation of oxidative stress defense genes, particularly upon H_2_O_2_ stress, and genes of the OxyR regulon were activated by putrescine in a concentration-dependent manner [34]. With respect to the dynamics of putrescine levels, our observations indicate that *E*. *coli* cells respond differently depending on the deployed ROS (e.g., H_2_O_2_ or HOCl) [22, 35].

### Changes in the composition of identifiable compounds of fatty acid metabolism

It has been previously shown that under HOCl stress, the cell envelope is strongly modified [22]. In particular, the expression of the outer membrane protein BhsA is induced under HOCl stress conditions, leading to a reduction of membrane permeability, subsequently increasing its hydrophobicity [25, 36]. Most fatty acids such as palmitic acid, palmitelaidic acid, pentadecanoic acid, and tetradecanoic acid (myristic acid) were not significantly affected by HOCl treatment for 20 min or longer (Fig 5). In contrast, the amount of stearic acid slightly decreased to 67% of its original level (40 min) but stabilized to prestressed levels after additional 20 min (101% after 60 min). Interestingly, metabolites assigned in the membrane structure and integrity, such as 1-monopalmitoylglycerol, 2-monopalmitoylglycerol, and 1-monooleoylglycerol, constantly increased over the observed 60 min. After 60 min, the concentrations reached levels of 182% (1-monopalmitoylglycerol), 167% (2-monopalmitolyglycerol), and 152% (1-monooleoylglycerol) (Fig 5). This could reflect cells producing new membrane compounds in an attempt to adapt to the oxidative stress [37]. The decrease in acetic acid levels within the first 20 min of HOCl exposure found by NMR analysis (shown above) could also indicate the increased consumption of acetate/acetyl-CoA for the synthesis of new fatty acids for supporting further growth. Moreover, increased levels of glycerol esters of fatty acids could benefit membrane integrity as a first line of defense when encountering ROS [14, 25, 38]. Notably, the levels of decanamide increased constantly over the entire 60 min (188% after 20 min; 193% after 40 min; 213% after 60 min), similar to the glycerol derivatives (Fig 5).

## Discussion

**Fig 6:**
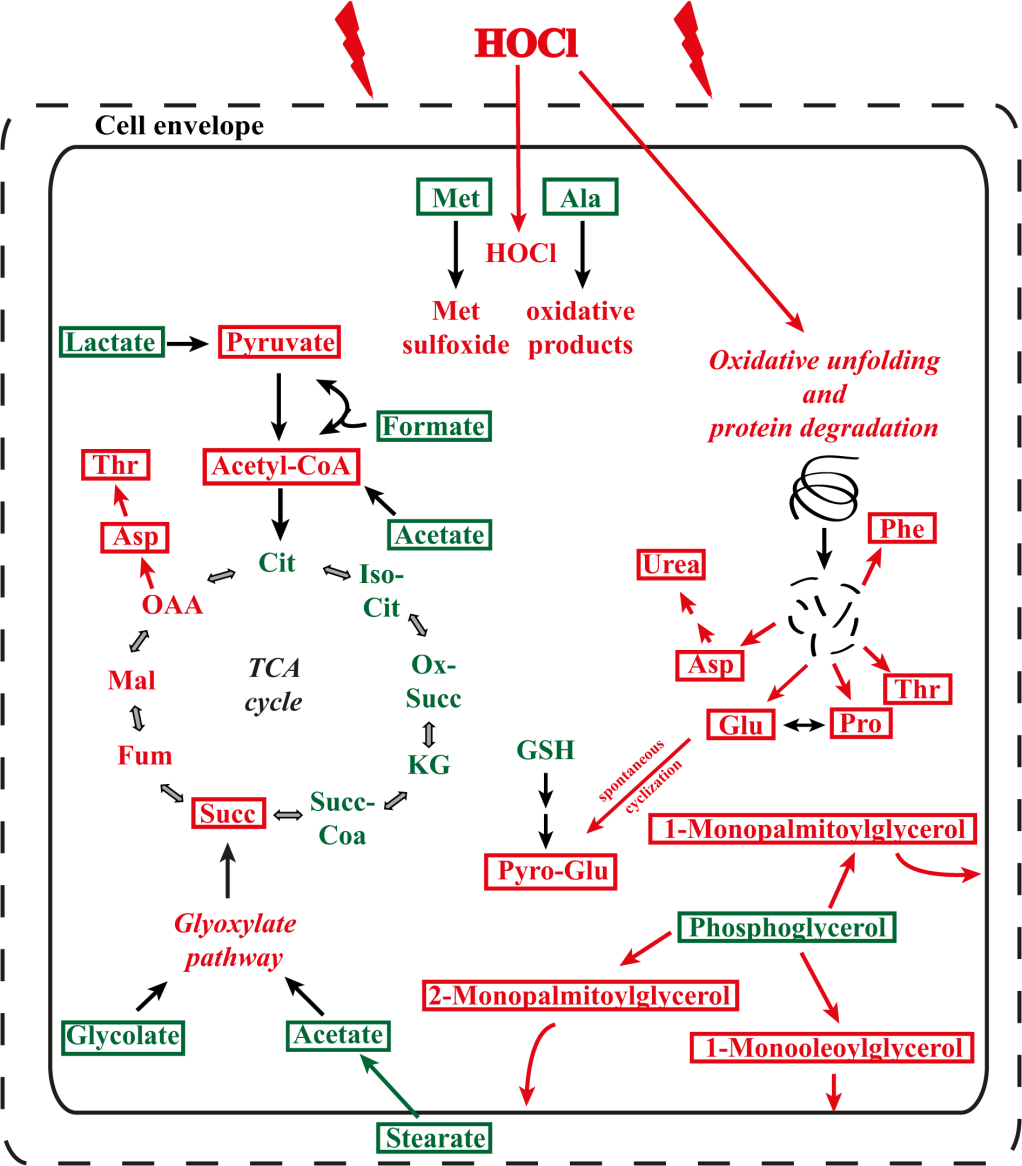
Model of metabolite alterations upon sub-lethal HOCl stress. Shown are the effects of HOCl stress within the first 20 min on the metabolism of *E*. *coli*. Identified metabolites are highlighted with a box. Metabolites that are downregulated are depicted in green, while upregulated metabolites are shown in red. Abbreviations: Ala, alanine; Asp, aspartate; Cit, citrate; Fum, fumarate; Glu, glutamate; GSH, glutathione; Iso-Cit, isocitrate; KG, α-ketoglutarate; Mal, malate; Met, methionine; Phe, phenylalanine; Pro, proline; Pyro-Glu, pyroglutamic acid; Succ, succinate; Succ-Coa, succinate-CoA; Ox-Succ, oxalosuccinate; Thr, threonine.

In the present study, we treated *E*. *coli* MG 1655 cells for 5–60 min with 50 μM HOCl. This concentration of HOCl was shown to be sub-lethal for this *E*. *coli* strain. We identified alterations in cellular metabolite levels by a combination of NMR and GC/MS analysis. We summarized our observations into a model (Fig 6). Earlier studies showed that oxidative stress results in a downregulation of central carbon pathways such as glycolysis and the tricarboxylic acid cycle (TCA cycle), whereas the glucose flux is re-directed to the NADPH-generating pentose phosphate pathway. The NADPH pool plays an important role in reductive detoxifying reactions [10, 23, 39–41]. This seems to be a common feature of oxidative stress, which is not limited to bacteria alone [42]. Most of these changes are generated by post-translational modifications of enzymes involved in glycolysis or the TCA cycle such as glyceraldehyde-3-phosphate dehydrogenase or aconitase [15, 22, 43]. Therefore, *E*. *coli* initiates the expression of ROS-resistant isoforms of important enzymes that are involved in the TCA cycle such as fumarate hydratase as a response to oxidative stress [25].

We showed that free alanine and methionine levels significantly decreased over time upon HOCl exposure. The decrease in methionine could result from the direct oxidation of methionine to methionine sulfoxide, which is a known mechanism of oxidative stress defense [27]. Thus, the decrease in methionine seems reasonable, although the transcription of methionine biosynthesis genes is upregulated [25]. A decrease in methionine levels was also demonstrated for H_2_O_2_ stress [23]. In the case of alanine, it is possible that it becomes directly oxidized by HOCl, which results in a conversion to acetaldehyde [44]. The generated acetaldehyde can then be detoxified by the NADPH-dependent aldehyde reductase YqhD, which was recently identified as a HOCl-regulated gene. YqhD particularly diminishes the negative effect resulting from lipid peroxidation, which ends up in the breakdown to short-chain aldehydes [33].

Several metabolites showed a similar regulation upon HOCl exposure as that described in a previous study upon H_2_O_2_ stress [23], e.g., phenylalanine, threonine, and phosphate (Table 2). Nevertheless, there are several metabolites that changed in a HOCl-specific manner, such as orotic acid, alanine, urea, succinate, and 2-hydroxybutyric acid (Table 2). This demonstrates that *E*. *coli* seems to react in a general manner for some metabolic pathways toward oxidative stress, whereas other pathways respond in a discriminative manner depending on ROS. Notably, significant alterations were detected in only 13 metabolites after 10 min of H_2_O_2_ exposure for *E*. *coli* cells in an exponential growth phase [23]. Most of these changes did not occur before 40 to 90 min [23]. It is likely that after this time period, additional factors had played roles in metabolite regulation, such as the growth phase and a biochemical switch from fermentation to respiration. In contrast, we observed more significant alterations as early as 5–20 min after HOCl stress (22 reproducibly identified metabolites were significantly altered by HOCl; Table 2). This demonstrates that HOCl exposure affects more metabolic pathways than H_2_O_2_ exposure. Nevertheless, several metabolites that were altered upon HOCl exposure returned to their prestressed levels within the observed hour (Table 2). This indicates that *E*. *coli* can adapt to HOCl stress by regenerating several altered metabolic pathways. The quick adaptation of *E*. *coli* cells to HOCl exposure and the reduction of oxidation products were also shown recently in the case of methionine sulfoxide and the action of the methionine sulfoxide reductases [18], complementing a previous study relating to H_2_O_2_ stress [23]. In addition, our analyses showed that the levels of fatty acids and glycerol esters of fatty acids were affected by HOCl stress. Fatty acids and compounds of the cell membrane are among the first components encountering HOCl molecules [45, 46]. It is a known mechanism that bacterial cells alter their membrane composition and cell envelope structure to antagonize HOCl stress, corroborating our observations [22, 38]. Besides the fatty acid composition, it was shown that the expression of several membrane-associated transport proteins such as BhsA and OmpW is affected by HOCl [25, 47]. Another metabolite identified by NMR was formic acid. The peak for formic acid significantly decreased after HOCl exposure. Formic acid is a key metabolite in energy metabolism and a major fermentation product of many enterobacteria [48, 49]. It is known that transcripts involved in aerobic metabolism are repressed by oxidative stress [25, 50]. HOCl exposure results in a downregulation of *pflB* (pyruvate formate-lyase) as well as *cydA* (cytochrome bd-I terminal oxidase subunit I) and *cydB* (cytochrome d terminal oxidase subunit II) [25]. All three genes are involved in aerobic metabolism. The downregulation of *pflB* could explain the observed decrease in the level of formic acid. One could hypothesize that low levels of formic acid together with the decreased level of lactic acid is the result of re-routing carbon fluxes for the production of pyruvate and acetyl-CoA (Fig 6). This could be a component of mechanisms that guarantee the storage of a minimal level of pyruvate, used for subsequent reactivation in the TCA cycle and sustained by newly expressed ROS-resistant isoforms such as *fumC* [25]. This would complement the observed increase in succinate, which is an important intermediate of the TCA cycle (Fig 6). Concomitantly, these HOCl-specific responses could ultimately benefit the regeneration of *E*. *coli* cells.

**Table 2:** Stress-dependent alteration of metabolite concentrations after 20 min as determined by GC/MS and comparison with H_2_O_2_ exposure.

Metabolite	Tendency after HOCl stress	Return to initial concentration	Tendency after H_2_O_2_ determined by Jozefczuk *et al*. [23]
**a) Metabolites that show similar behavior upon HOCl and H**_**2**_ **O**_**2**_ **stress**			
Aspartate	↑	**yes**	↑
Glutamate	↑	**yes**	↑
Phenylalanine	↑	**yes**	↑
Threonine	↑	**yes**	↑
Pyroglutamic acid	↑	no	↑
**b) Metabolites that do not alter upon both stress conditions**			
Proline	Ø	no	Ø
Putrescine (1,4-Diaminobutane)	Ø	no	Ø
Pelargonic acid	Ø	no	Ø
Palmitic acid (Decanoic acid)	Ø	no	Ø
Palmitelaidic acid (9-trans Decenoic acid)	Ø	no	Ø
**c) Metabolites that show different alterations or were not identified by Jozefcuk *et al*.**			
Succinate (Butanedioic acid)	↑	**yes**	Ø
Urea	↑	**yes**	Ø
Phosphate	↑	no	Ø
Decanamide (Capramide)	↑	no	n.d
1-Monopalmitoylglycerol	↑	no	n.d
2-Monopalmitoylglycerol	↑	no	n.d
1-Monooleoylglycerol	↑	no	n.d
Glycine	Ø	no	↑
Leucine	Ø	no	↓
Valine	Ø	no	↓
Tyrosine	Ø	no	↑
Tetradecanoic acid (Myristic acid)	Ø	no	n.d
Pentadecanoic acid	Ø	no	n.d
Alanine	↓	no	↑
Methionine	↓	no	Ø
Lactic acid	↓	no	n.d
2-Aminobutyric acid	↓	no	↑
Pyrimidine	↓	**yes**	n.d
Phosphoglycerol	↓	**yes**	Ø
2-Hydroxybutyric acid	↓	no	Ø
Orotic acid	↓	no	Ø
Glycolic acid	↓	no	Ø
Stearic acid (Octadecanoic acid)	↓	**yes**	Ø

Overview of the concentration alterations after HOCl exposure of metabolites included in the discussion of the main text. Comparison of metabolite levels of a previous study by Jozefczuk et al. who analyzed the metabolite alteration after H_2_O_2_ exposure. Alterations of the concentrations are indicated by arrows (↑ increase; ↓ decrease). For detailed information, see S2 Table.

## Supporting Information

S1 Fig Typical ^1^H-NMR spectra of extracts from stressed and unstressed cells. Typical 500 MHz ^1^H-NMR spectra of D_4_-methanol extracts of unstressed *E*. *coli* MG1655 cells (black) and cells stressed for 5 min (blue), 10 min (green), 20 min (red), 40 min (orange), and 60 min (purple).
(TIF)

S2 Fig Typical chromatogram of a GC run. Typical GC chromatogram of an unstressed control sample. Several identified metabolites are assigned to their corresponding peaks.
(TIF)

S1 Table Number of samples integrated into principal component analysis. The experimental condition “no HOCl stress” was independently repeated seven times (seven biological replicates) and each biological replicate was measured three times (three technical replicates), yielding 21 spectra for the control group. For the 5 min stress treatment, 28 spectra were recorded, resulting from 18 biological replicates, five of which were measured three times. For the 10 min stress treatment, 19 spectra from seven biological repetitions were acquired, with six out of seven samples being measured three times. For the 20 min stress treatment, 18 spectra were obtained from six biological replicates, each being measured three times. For the 40 min and 60 min stress treatment, six biological replicates were conducted, each being measured once. Some samples were measured three times to prove the validity and stability of NMR measurement.
(PDF)

S2 Table Retention times and typical mass peaks of compounds identified by GC/MS analysis in extracts of untreated control cells (41 mg of dry cell mass). List of peaks from a GC chromatogram of an extract of unstressed *E*.*coli* cells (40.7 mg dry cell mass). Peaks are ordered chronologically with increasing retention times. The list also includes the information of the typical m/z mass peaks, the relative concentrations of the corresponding peaks, their most probable and reliable identities, and the similarity of the mass peaks compared with the NIST05 and NIST05s reference library in percentage.
(PDF)

S3 Table Peaks originating from unidentified compounds that are not illustrated and/or discussed in the main text (Figs 4–6). Shown are the retention times of peaks listed in S2 Table but not shown in Figs 4–6 of the main text and their mean concentrations ± SD before stress and after 20 min, 40 min, and 60 min of HOCl exposure (n ≥ 3).
(PDF)
