# Supplementary figures and images for: Metabolic Response of Escherichia coli upon Treatment with Hypochlorite at Sub-Lethal Concentrations
Source: PLoS One. 2015 May 1;10(5):e0125823. doi: 10.1371/journal.pone.0125823 (PMC4416902; doi:10.1371/journal.pone.0125823)

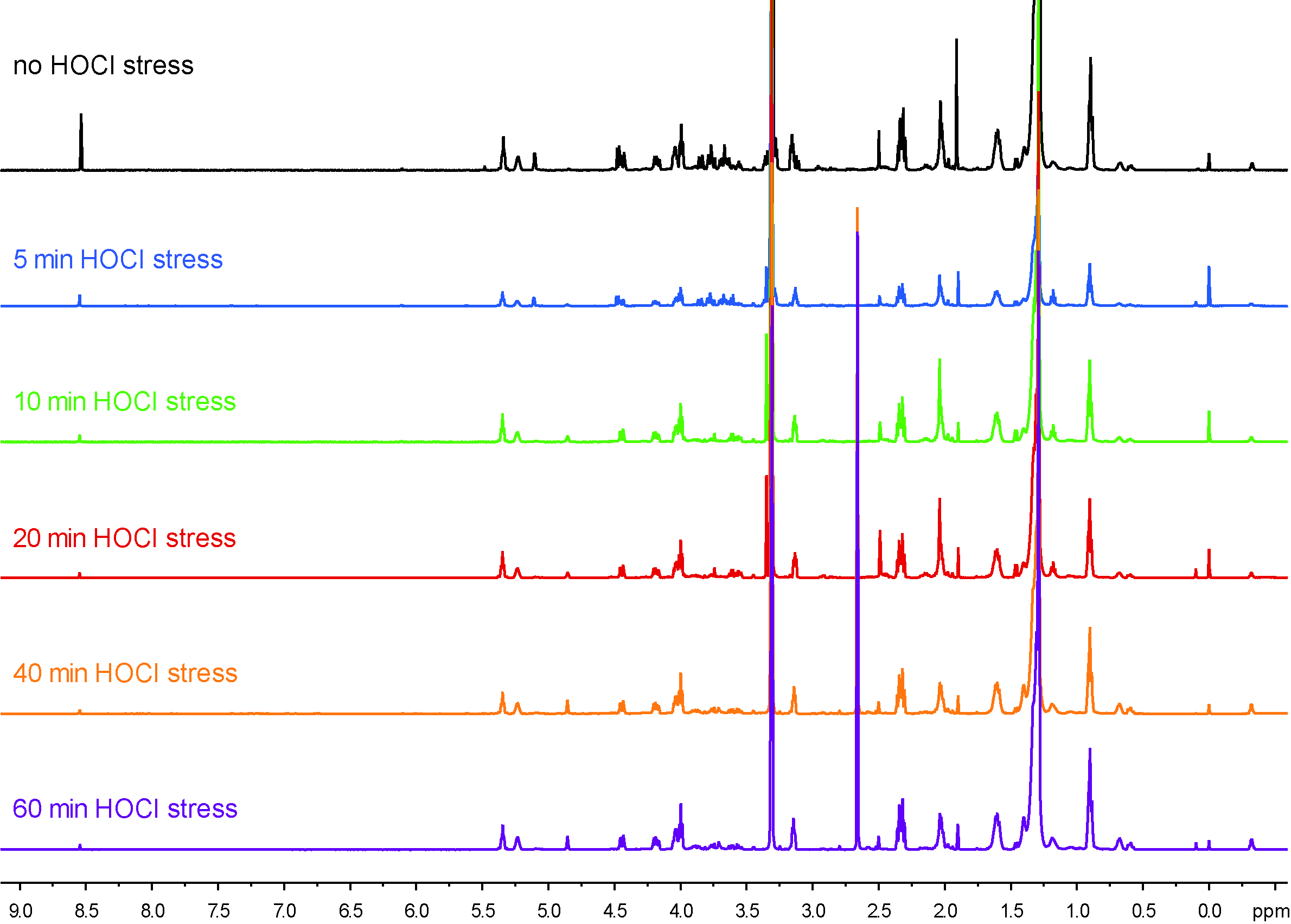

Supplement: S1 Fig — Typical 500 MHz 1H-NMR spectra of D4-methanol extracts of unstressed E. coli MG1655 cells (black) and cells stressed for 5 min (blue), 10 min (green), 20 min (red), 40 min (orange), and 60 min (purple). (TIF) [file pone.0125823.s001.tif]

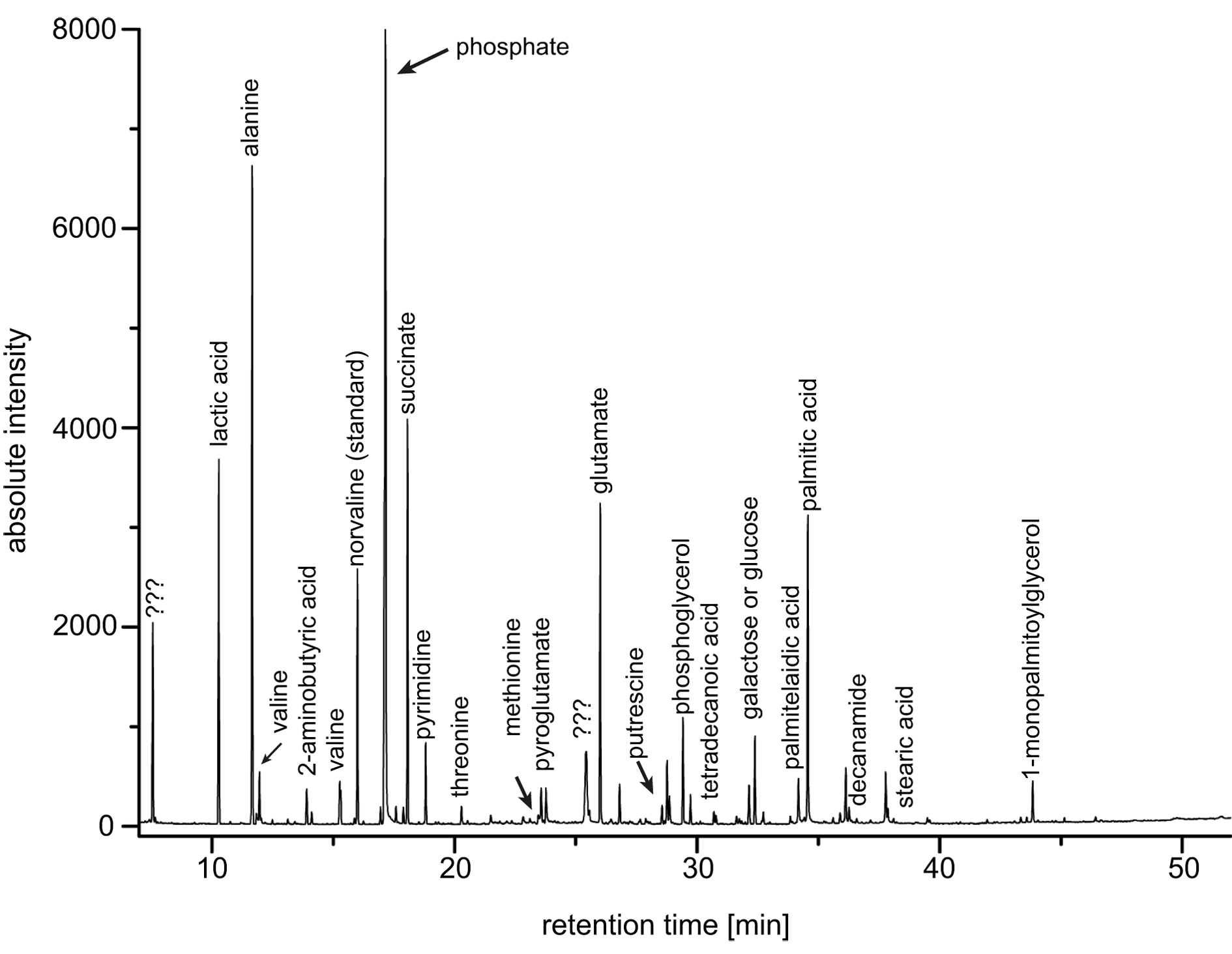

Supplement: S2 Fig — Typical GC chromatogram of an unstressed control sample. Several identified metabolites are assigned to their corresponding peaks. (TIF) [file pone.0125823.s002.tif]
