# Supplementary material for: Metabolic Response of Escherichia coli upon Treatment with Hypochlorite at Sub-Lethal Concentrations
Source: PLoS One. 2015 May 1;10(5):e0125823. doi: 10.1371/journal.pone.0125823 (PMC4416902; doi:10.1371/journal.pone.0125823)
Supplement: S1 Table — The experimental condition “no HOCl stress” was independently repeated seven times (seven biological replicates) and each biological replicate was measured three times (three technical replicates), yielding 21 spectra for the control group. For the 5 min stress treatment, 28 spectra were recorded, resulting from 18 biological replicates, five of which were measured three times. For the 10 min stress treatment, 19 spectra from seven biological repetitions were acquired, with six out of seven samples being measured three times. For the 20 min stress treatment, 18 spectra were obtained from six biological replicates, each being measured three times. For the 40 min and 60 min stress treatment, six biological replicates were conducted, each being measured once. Some samples were measured three times to prove the validity and stability of NMR measurement. (PDF) [file pone.0125823.s003.pdf]

**S1 Table. Number of samples integrated into Principal Component Analysis.**

| Treatment with HOCl | Number of biological replicates | Number of $^{1}H$ NMR measurements | Total number of $^{1}H$ NMR spectra |
|---|---|---|---|
| **No HOCl** | 7 | 3 | 21 |
| **5 min exposure** | 13<br>5 | 1<br>3 | 28 |
| **10 min exposure** | 6<br>1 | 3<br>1 | 19 |
| **20 min exposure** | 6 | 3 | 18 |
| **40 min exposure** | 6 | 1 | 6 |
| **60 min exposure** | 6 | 1 | 6 |

The experimental condition "no HOCl stress" was independently repeated seven times (seven biological replicates) and each biological replicate were measured three times (three technical replicates), yielding 21 spectra for the control group. For the 5 min stress treatment 28 spectra were recorded, they resulted from 18 biological replicates, five of which were measured three times. For the 10 min stress treatment 19 spectra from seven biological repetitions were gained, six out of seven samples were measured three times. For the 20 min stress treatment 18 spectra were obtained from six biological replicates, each measured three times. For the 40 min and 60 min stress treatment six biological replicates were conducted, each measured once. Some samples were measured three times in order to prove the validity and stability of the NMR measurement.
