# Supplementary material for: Metabolic Response of Escherichia coli upon Treatment with Hypochlorite at Sub-Lethal Concentrations
Source: PLoS One. 2015 May 1;10(5):e0125823. doi: 10.1371/journal.pone.0125823 (PMC4416902; doi:10.1371/journal.pone.0125823)
Supplement: S2 Table — List of peaks from a GC chromatogram of an extract of unstressed E.coli cells (40.7 mg dry cell mass). Peaks are ordered chronologically with increasing retention times. The list also includes the information of the typical m/z mass peaks, the relative concentrations of the corresponding peaks, their most probable and reliable identities, and the similarity of the mass peaks compared with the NIST05 and NIST05s reference library in percentage. (PDF) [file pone.0125823.s004.pdf]

S2 Table. Retention times and typical mass peaks of compounds identified by GC/MS analysis in extracts of untreated control cells (40.7 mg dry cell mass).

**Peak #**	**Retention time [min]**	**Typ. Mass peaks [m/z]**	**Integrated peak area**	**ID [NIST05 library]**	Similarity target/library **[%]**
**1**	7.560	69, 90, 140, 187	4867829	M-Methyl-N-n-butyltrifluoroacetamide	75
**2**	7.657	75, 113, 155, 170	136686	3-Methy-1-cyclopenten-1yl	78
**3**	10.284	88, 117, 191, 219	8707496	Lactic acid	97
**4**	10.755	103, 133, 177, 210	55352	Glycolic acid	92
**5**	11.661	100, 0116, 190, 218	16491873	Alanine	98
**6**	11.836	103, 128, 156, 233	373752	Valine	91
**7**	11.960	117, 147, 148, 207	1350507	Oxalic acid	82
**8**	12.032	55, 75, 103, 137, 138, 167, 169, 211	133108	4-Bromobutanol	81
**9**	12.493	131, 190, 205 ,233	108092	2-Hydroxybutyric acid	95
**10**	13.131	59, 100, 130, 174 208	119095	2-Chloro-2-oxo-Trieethylamine	92
**11**	13.908	100, 130, 131, 204 ,218	900856	2-Aminobutyric acid	94
**12**	15.265	100, 114, 128, 144, 147	1169106	Valine	95
**13**	15.306	59, 100, 130, 174	667491	Ethylbisamine	88
**14**	15.877	99, 189, 204	157853	Urea	90
**15**	15.999	100, 133, 144, 174	6148501	Norvaline (standard)	88
**16**	16.951	102, 158, 218, 232, 260	424505	Isoleucine	93
**17**	17.152	103, 133, 207, 211, 299	30759403	Phosphate	93
**18**	17.591	100, 142, 175, 216, 244	315139	Proline	96
**19**	17.895	174, 248, 276	405973	Glycine	90
**20**	18:066	172, 218, 247	9436933	Succinate	90
**21**	18.531	98, 135, 151, 195, 211, 252	55698	3-Aminopropylphosphonic acid	71
**22**	18.733	189, 217, 292	38161	Glyceric acid	78
**23**	18.814	99, 113, 241	2002235	Pyrimidine	93
**24**	19.225	75, 81, 117, 132, 145, 172, 215	42396	Nonanoic acid (Pelargonic acid)	85
**25**	19.341	100, 113, 147, 188, 189, 241, 156	55939	2,5-Dioxypyrazine	75
**26**	20.286	117, 180, 219, 291, 320	511942	Threonine	90
**27**	20.538	100, 113, 147, 255, 270	87521	5-Methylpyrimidine	89
**28**	22.153	77, 107, 179, 200, 255, 270	111994	2-oxy-Ethanesulfonic acid	72
**29**	22.358	105, 136, 179, 193	80421	3-Pyridinecarboximidoate	75
**30**	22.829	84, 116, 157, 186, 255	280692	2-Piperidinecarboxylic acid	79
**31**	23.111	111, 172, 217, 275	118464	Hexanedioic acid (Adipic acid)	88
**32**	23.509	61, 100, 128, 176, 219	156530	Methionine	80
**33**	23.574	112, 156, 130 ,258	1033594	Pyroglutamic acid	95

**34**	23.665	59, 100, 117, 133, 218, 232	73358	Aspartic acid	84
**35**	23.772	84, 174, 230, 276, 304	1148157	N-acetyl-glutamic acid	73
**36**	25.560	110, 147, 179, 239, 327 ,342	340276	1,2,3-Trioxybenzene	84
**37**	26.011	56, 100, 128, 174, 204, 230, 246, 258, 320, 348	7867583	Glutamic acid	89
**38**	27.225	112, 274, 299, 451, 467	104024	Pyrophosphate	73
**39**	27.600	83, 129, 187, 303	60185	Octanedioic acid (Suberic acid)	81
**40**	27.661	103, 189, 217, 263, 307	109757	O-methyloxime-d-Ribose	86
**41**	27.887	103, 183, 255, 345	177411	Ethanesulfonic acid	71
**42**	28.556	100, 130, 174, 214, 361	517042	1,4-Butanediamine (Putrescine)	92
**43**	28.655	103, 129, 211, 243, 299, 389, 445	76568	Phosphoric acid	88
**44**	28.863	100, 174, 214, 254, 271, 329, 357	1708657	4-Pyrimidinecarboxylic acid (Orotic acid)	94
**45**	29.418	211, 256, 299, 315, 357, 370, 445	2535810	Phospho-propylester	96
**46**	29.731	114, 174, 188, 299, 315, 414	757431	Phospho-aminoethylester	87
**47**	30.136	116, 173, 216, 290, 318	74199	Caproic acid	70
**48**	30.693	117, 129, 132, 145, 201, 285	364886	Tetradecanoic acid	95
**49**	30.785	117, 147, 191, 218	238092	Tris-O-d-Erythrotetrofuranose	80
**50**	31.625	91, 146, 179, 208, 310	231117	Tyrosine	87
**51**	31.745	83, 98, 123, 152, 194, 236	53668	13-Docosenoic acid (Brassidic acid)	80
**52**	31.841	103, 147, 189, 217, 277, 307, 364	86437	Fructose	88
**53**	31.986	103, 129, 189, 220, 243, 319, 361	61600	Mannonic acid-lactone	88
**54**	32.382	103, 147, 205, 319	2252347	Galactose / Glucose	95
**55**	32.670	117, 129, 132, 201, 255, 299	77708	Pentadecanoic acid	84
**56**	32.732	103, 147, 205, 229, 291, 319	313324	Glucose/Galactose	94
**57**	34.178	75, 96, 117, 129, 152, 194, 236, 311	1563624	Palmitelaidic acid	95
**58**	34.419	85, 103, 147, 236, 292, 333, 359, 423	235155	D-Gluconic acid	86
**59**	34.565	83, 117, 129, 132, 145, 201, 313	8568088	Palmitic acid	95
**60**	36.131	75, 81, 96, 117, 189, 199, 325	1746756	Oleic acid	89
**61**	36.267	85, 98, 112, 140 ,196	502019	N-2-Hydroxyethyl-Decanamide	95
**62**	37.778	75, 117, 129, 185, 222, 264, 339	1582251	11-cis-Octadecenoic acid	96
**63**	38.100	75, 117, 129, 132, 201, 341	186963	Stearic acid	93
**64**	39.495	85, 86, 95, 112, 126, 140	174278	Hydoxyethylpalmitamide	76
**65**	43.344	103, 129, 147, 191, 218, 239, 313	149875	2-Monopalmitin	91
**66**	43.592	103, 129, 147, 203, 237, 369, 398	143281	1-Monooleoylglycerol	85
**67**	43.839	103, 129, 147, 203, 239, 313, 371	1195412	1-Monopalmitin	94

List of peaks from a GC chromatogram of an unstressed control sample (40.7 mg dry cell mass). Peaks are ordered chronologically with increasing retention times. The list also includes the information of the typical m/z mass peaks, the relative concentrations of the corresponding peaks, their most probable and reliable identities, and the similarity of the mass peaks compared to the NIST05 mass library in percentage.
