# Supplementary material for: Metabolic Response of Escherichia coli upon Treatment with Hypochlorite at Sub-Lethal Concentrations
Source: PLoS One. 2015 May 1;10(5):e0125823. doi: 10.1371/journal.pone.0125823 (PMC4416902; doi:10.1371/journal.pone.0125823)
Supplement: S3 Table — Shown are the retention times of peaks listed in S2 Table but not shown in Figs 4–6 of the main text and their mean concentrations ± SD before stress and after 20 min, 40 min, and 60 min of HOCl exposure (n ≥ 3). (PDF) [file pone.0125823.s005.pdf]

S3 Table. Peaks originating from unidentified compounds and not illustrated and/or discussed in the main text (Fig. 4-6).

	control		20 min HOCl		40 min HOCl		60 min HOCl	
Ret. Time	mean	SD	mean	SD	mean	SD	mean	SD
7,56	192859,58	103600,91	133797,04	85267,74	42126,54	11145,50	43540,35	9994,87
7,66	3400,12	59,04	3529,40	123,49	3028,70	284,59	2887,04	132,80
11,96	21578,21	16410,22	17220,74	9073,92	13227,15	3817,84	22840,80	18461,80
12,03	4128,25	1213,09	3736,10	433,31	3533,97	413,68	2907,88	470,42
13,13	3662,94	1041,95	3708,63	1484,48	4738,27	2862,76	14230,35	18972,52
14,12	6205,08	2616,96	5022,88	986,73	5579,45	231,74	6277,47	1410,49
16,00	152223,18	1632,51	172938,46	14689,51	178442,79	13256,80	146962,42	38270,33
16,25	1802,67	777,83	1031,68	59,51	799,97	65,16	516,02	194,81
18,53	1707,98	480,09	3619,89	1551,93	7159,42	4582,55	7158,83	5332,18
18,73	908,32	41,43	992,16	197,16	892,20	74,80	877,65	163,55
21,50	4395,24	1732,59	3027,86	1072,11	3014,65	970,97	3488,74	1266,08
21,87	1356,40	297,94	2023,93	336,46	2465,52	632,39	2520,15	890,19
22,15	2175,92	814,28	1368,12	318,18	866,92	474,77	833,67	168,73
22,36	2035,90	84,79	2932,22	415,61	3562,16	440,17	3558,81	655,75
22,83	5426,46	2079,11	6395,77	1016,38	5339,83	48,40	5671,54	1560,15
23,11	2305,93	855,22	1135,90	851,78	709,90	182,00	720,96	332,77
23,45	5924,96	151,88	2027,95	890,75	1406,99	303,43	1736,35	307,78
23,77	25054,44	4462,99	36291,41	5140,88	26030,47	805,03	36451,45	21421,82
25,43	88973,20	30636,23	73515,89	11073,19	67122,91	6035,86	66698,05	14815,30
25,56	8065,07	417,93	11241,18	913,68	14076,44	2330,85	11753,94	2746,10
26,25	1177,04	601,60	1019,44	327,73	1109,03	232,30	1086,84	255,73
26,45	5619,66	1037,09	7033,55	1022,33	5495,42	1898,03	4923,13	2572,33
26,81	23117,12	3309,51	5459,27	579,17	2273,49	388,16	1259,93	234,39
27,23	2942,92	547,38	764,66	204,01	2047,73	589,27	2092,26	450,01

**27,60**	**1569,10**	**127,78**	**1580,09**	**831,58**	**622,81**	**111,87**	**499,18**	**249,26**
**27,66**	**2375,54**	**454,24**	**2621,25**	**1097,40**	**2568,60**	**247,64**	**2307,97**	**253,07**
**27,89**	**3300,27**	**1497,26**	**2092,49**	**264,25**	**1501,88**	**389,54**	**472,37**	**151,77**
**27,97**	**1446,75**	**393,14**	**1429,15**	**351,40**	**1270,71**	**234,04**	**1645,19**	**554,48**
**28,06**	**974,14**	**517,40**	**765,88**	**80,04**	**988,68**	**127,79**	**1084,78**	**327,22**
**28,66**	**1505,12**	**531,97**	**1037,81**	**167,66**	**1437,67**	**108,21**	**3608,14**	**3130,11**
**28,77**	**36248,17**	**8108,50**	**26823,28**	**4354,41**	**33707,18**	**3805,79**	**30659,83**	**6437,91**
**29,42**	**44822,34**	**24724,09**	**17698,27**	**1209,96**	**34170,08**	**1599,56**	**32734,59**	**7259,01**
**29,73**	**11718,56**	**9746,10**	**2071,42**	**527,11**	**5542,08**	**1315,29**	**7047,14**	**2369,00**
**29,98**	**884,51**	**137,51**	**477,34**	**313,79**	**2805,85**	**1315,11**	**1292,16**	**1031,34**
**30,14**	**2768,01**	**1336,35**	**811,36**	**209,53**	**1014,25**	**90,48**	**390,56**	**348,42**
**30,69**	**8542,20**	**598,29**	**8356,15**	**625,11**	**9622,17**	**760,47**	**10173,24**	**2123,40**
**30,79**	**5931,02**	**114,69**	**3519,70**	**974,30**	**1958,44**	**185,52**	**1657,07**	**945,61**
**31,75**	**1998,28**	**961,17**	**2894,65**	**865,68**	**2780,89**	**732,01**	**2216,62**	**1119,36**
**31,84**	**1982,54**	**199,71**	**2548,57**	**593,47**	**1726,91**	**229,16**	**1013,91**	**511,85**
**31,99**	**1193,00**	**453,28**	**869,93**	**414,00**	**698,20**	**301,67**	**416,53**	**143,20**
**32,15**	**24460,09**	**6705,06**	**26484,53**	**6853,64**	**35537,34**	**9119,14**	**35271,80**	**5415,69**
**32,38**	**46427,87**	**12603,97**	**70549,17**	**52104,25**	**28571,86**	**13334,55**	**12896,14**	**5346,27**
**32,73**	**6100,81**	**2259,30**	**8261,71**	**5869,70**	**3311,73**	**1963,28**	**2013,63**	**427,40**
**34,42**	**5143,47**	**897,03**	**3034,82**	**915,68**	**1925,10**	**249,25**	**2137,04**	**397,17**
**35,24**	**1292,85**	**--**	**1305,07**	**542,36**	**1217,35**	**245,30**	**1817,10**	**1222,74**
**36,27**	**21738,69**	**13299,37**	**38353,05**	**5661,84**	**30214,30**	**19095,69**	**41199,11**	**5668,61**
**37,15**	**1320,93**	**647,80**	**875,53**	**274,85**	**702,53**	**422,90**	**833,71**	**157,48**
**37,78**	**36799,71**	**2936,24**	**39074,94**	**3376,05**	**40520,99**	**4810,69**	**46901,49**	**11736,95**
**39,50**	**8013,73**	**5277,45**	**11996,25**	**2239,13**	**11109,09**	**942,88**	**11664,08**	**5189,10**
**41,94**	**3377,08**	**1331,93**	**4285,26**	**2216,27**	**3432,15**	**493,25**	**3517,61**	**1058,85**

Shown are the retention times of peaks listed in Table S2, but not shown in Figures 4-6 of the main text, and their mean concentrations ± SD before stress and after 20 min, 40 min and 60 min HOCl exposure ($n \geq 3$).
